# Comparison of Heart Failure Hospitalizations with and Without Respiratory Syncytial Virus: A Nationwide Administrative Data Analysis

**DOI:** 10.3390/jcm15030990

**Published:** 2026-01-26

**Authors:** Nikita Patil, Shubhadarshini Pawar, Lakshmi Menon, Prasad Jogu, Sagar Bathija, Mahita Bellamkonda, Muskan Joshi, Swathi Nimmala, Arun R. Sridhar

**Affiliations:** 1 Hospitalist Department, Cape Fear Valley Health, Fayetteville, NC 28304, USA; swathireddy.nimmala@gmail.com; 2 Department of Cardiology, Smidt Heart Institute, Cedars Sinai Medical Center, Los Angeles, CA 90048, USA; 3 Division of Endocrinology and Metabolism, Department of Internal Medicine, University of Arkansas for Medical Sciences, Little Rock, AR 72205, USA; lpmenon@uams.edu; 4 Hospitalist Department, Sutter Roseville Medical Center, Roseville, CA 95661, USA; drprasadgmc@gmail.com; 5 Department of Internal Medicine, Lowell General Hospital, Tufts Medicine, Lowell, MA 01854, USA; sagar.bathija27@gmail.com; 6 Department of Hospital Medicine, Oklahoma University Health Science Center, Oklahoma City, OK 73104, USA; docmahitab@gmail.com; 7 International Faculty of Medicine, Tbilisi State Medical University, Tbilisi 0186, Georgia; 8 Department of Cardiology, Pulse Heart Institute, Multicare Health System, Tacoma, WA 98402, USA; dr.arunraghav@gmail.com

**Keywords:** mortality, respiratory failure, septic shock, healthcare utilization, readmissions, length of stay

## Abstract

**Background:** Heart failure (HF) remains a major cause of hospitalizations in the United States (US). Respiratory syncytial virus (RSV) has been associated with HF exacerbations. We compared in-hospital outcomes and healthcare utilization among US HF hospitalizations with and without RSV. **Methods:** Using the Nationwide Readmissions Database (2016–2022), we propensity-matched HF hospitalizations with a secondary diagnosis of RSV (HF-RSV) 1:1 to those without RSV (HF-noRSV). Multivariable logistic and Poisson regression models were used to assess associations between RSV and outcomes. The primary outcome was in-hospital mortality; secondary outcomes included adverse events, length of stay (LOS), hospitalization costs, and 30-day readmissions. **Results:** Among 11,158,836 HF hospitalizations, 32,419 (0.29%) had RSV. Compared with matched HF-noRSV hospitalizations, HF-RSV was associated with higher odds of in-hospital mortality (adjusted odds ratio [aOR] 1.12; 95% CI 1.04–1.20), septic shock (aOR 1.40; 95% CI 1.29–1.52), acute respiratory failure (aOR 3.43; 95% CI 3.32–3.55), and noninvasive mechanical ventilation (aOR 2.15; 95% CI 2.04–2.26). HF-RSV had lower odds of cardiogenic shock (aOR 0.82; 95% CI 0.73–0.92), ventricular tachycardia/fibrillation (aOR 0.73; 95% CI 0.68–0.78), ischemic stroke (aOR 0.31; 95% CI 0.27–0.36), transient ischemic attack (aOR 0.33; 95% CI 0.25–0.44), and 30-day readmissions (aOR 0.54; 95% CI 0.46–0.56). HF-RSV hospitalizations had higher costs (adjusted coefficient 0.02; 95% CI 0.01–0.02) and longer LOS (adjusted coefficient 0.14; 95% CI 0.13–0.14). **Conclusions:** Among US HF hospitalizations, RSV was associated with higher mortality and respiratory-related complications and increased healthcare resource utilization.

## 1. Introduction

Respiratory syncytial virus (RSV) is a seasonal respiratory pathogen that can cause severe lower respiratory tract infections in adults, often necessitating hospitalization. In the United States (US), RSV accounts for approximately 160,000 hospitalizations and more than one million outpatient visits annually among older adults [1], contributing to an estimated economic burden of 1.3 billion dollars [2]. Among hospitalized adults, the incidence of RSV is higher in those with heart failure (HF), as demonstrated in a multicenter New York study [3]. Furthermore, nearly one-quarter of adults hospitalized with RSV experience acute cardiac complications, including HF exacerbation, arrhythmias, myocarditis, and acute coronary syndromes [4].

HF itself remains a major public health challenge in the US, affecting 6.7 million adults and accounting for 1.2 million hospitalizations in 2021 [5]. Despite advances in therapy, nearly one in five patients are readmitted within 30 days [6]. Viral infections such as influenza [7] and cytomegalovirus [8] have been associated with increased mortality in hospitalized HF patients, yet data regarding the impact of RSV in this population remain limited.

A previous study suggested that HF patients have an eight-fold increased risk of RSV-associated hospitalization [9], and a recent national analysis from Spain reported that RSV in HF hospitalizations was associated with higher in-hospital mortality, greater need for noninvasive ventilation, and increased length and cost of stay [10]. However, contemporary data describing the burden and outcomes of RSV among HF hospitalizations in the US are lacking. Accordingly, we used the Nationwide Readmissions Database (NRD) to evaluate the association between RSV infection and in-hospital outcomes among adults hospitalized with a primary diagnosis of HF in the US.

## 2. Methods

### 2.1. Data Source

NRD, a component of the Healthcare Cost and Utilization Project sponsored by the Agency for Healthcare Research and Quality, provides comprehensive data on hospital admissions across the US [11]. This dataset contains about 60% of all hospitalizations in the US and is derived from 84% of discharges recorded in State Inpatient Databases, with no restrictions on payor status. This study used data from 1 January 2016 to 31 December 2022. Unweighted, it comprises information from around 7 million hospital stays per year [11]. The dataset records readmissions using patient linkage information to identify the same person across hospitals in a state within a calendar year. Because the dataset was publicly available and de-identified, the Institutional Review Board’s approval and informed consent were not required.

### 2.2. Study Population

**Figure 1:**
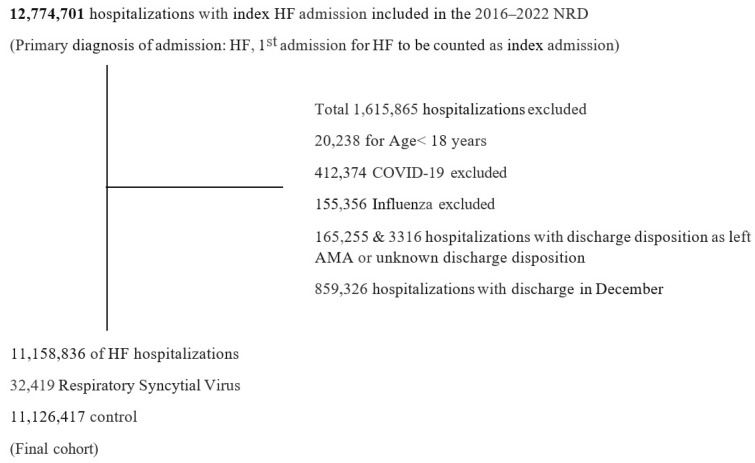
Study population and selection criterion. Among 12,774,701 index heart failure (HF) hospitalizations identified from the Nationwide Readmissions Database (NRD) from 2016 to 2022, a total of 11,158,836 were included in the final cohort. Of these, 32,419 hospitalizations had a secondary diagnosis of Respiratory Syncytial Virus. Exclusion criteria were based on age < 18 years, the presence of COVID-19 or influenza, discharge disposition unknown or against medical advice (AMA), or discharge in December. NRD linkage does not allow patients to be tracked across calendar years. Therefore, hospitalizations with discharge in December were excluded to account for the 30-day readmission window.

We incorporated index HF hospitalizations of adult patients (≥18 years) during the calendar year, utilizing the International Classification of Diseases, Tenth Revision, and Clinical Modification (ICD 10-CM) codes (Supplemental Table S1). The index hospitalization was defined as the first HF hospitalization of the calendar year. Because NRD does not allow for year-to-year linkage, patients and hospitals from each year were treated separately. We excluded hospitalizations (1) with discharge disposition as left against medical advice or unknown discharge disposition, (2) with COVID-19 or influenza, and (3) that occurred in December (to allow for assessment of 30-day readmissions). This yielded a final study population of 11,158,836 HF hospitalizations. We further stratified HF hospitalizations into ‘with’ and ‘without’ RSV (ICD 10-CM: B974, J121, J205, J210) as a secondary diagnosis (Figure 1). Baseline characteristics of the hospitalizations were identified using ICD-10-CM, ICD-10-PCS (Procedure Classification System), and the Elixhauser comorbidity software (Supplemental Table S1).

### 2.3. Study Outcomes

The primary outcome of this study was in-hospital mortality. Secondary outcomes included septic shock, cardiogenic shock, acute coronary syndrome, ventricular tachycardia/ventricular fibrillation, cardiac arrest, acute respiratory failure, invasive mechanical ventilation, noninvasive mechanical ventilation, ischemic stroke, transient ischemic attack (TIA), acute renal failure, discharge to a skilled nursing facility, 30-day all-cause readmissions, length of stay (LOS) and cost of hospitalization.

### 2.4. Statistical Analysis

Statistical analysis was performed using Stata, version 18 (Statistical Software: Release 18. College Station, TX, USA: Stata Corp LLC). Categorical data were reported as frequency (%), whereas continuous variables were represented as median (interquartile range). A standardized mean difference (SMD) of 0.2–0.5, 0.5–0.8, and >0.8 represents a small, medium, and large effect size, respectively. Variables included in the multivariable logistic regression model were selected based on statistical relevance and clinical importance. Candidates for inclusion were initially identified through univariable analysis using a liberal threshold of *p* < 0.20. To assess multicollinearity, we calculated the Variance Inflation Factor (VIF) for all predictors, and none exceeded the commonly accepted cutoff of 10, indicating no concerning collinearity. Then, 1:1 propensity score matching was performed to account for non-random treatment assignment (with RSV vs. no RSV). Propensity scores were estimated using a non-parsimonious multivariable logistic regression model adjusting for the following covariates—age, sex, primary expected payor, median household income, calendar year of admission, comorbidities including smoking, dyslipidemia, diabetes, hypertension, obesity, known coronary artery disease, prior MI, prior PCI, prior coronary artery bypass grafting, prior TIA/stroke, peripheral vascular disease, anemia, chronic kidney disease, chronic lung disease, chronic liver disease, coagulopathy, hypothyroidism, pulmonary circulation disorders, cancer, hospital characteristics such as bed size, urban vs. rural location, teaching vs. nonteaching status (Table 1). Model selection was further refined using the Akaike Information Criterion (AIC) and Bayesian Information Criterion (BIC), allowing us to balance model fit with parsimony. Secondary outcomes, such as cost and length of stay, were analyzed using Poisson regression models, which were adjusted for the same covariates as the multivariable logistic regression model. We did not aim to obtain national estimates. Therefore, unweighted data were employed for analysis, consistent with prior studies on hospital-level estimations [12,13]. The weighted estimates in the NRD are for discharge-level data and do not apply to hospital-level studies after the 2012 database update [14]. The dataset’s complex survey design was addressed with clustering and stratification variables, as recommended by the Agency for Healthcare Research and Quality Methods Series [15]. Missing data were addressed using simple imputation, utilizing the predominant category for categorical variables and the median value for continuous variables. The incidence of missing data was minimal: 0.12% for payor status, 1.24% for median household income quartile, 0.37% for hospital location, and 0.03% for mortality status. All *p*-values were two-sided with a significance threshold of <0.05. The study follows the Strengthening the Reporting of Observational Studies in Epidemiology (STROBE) reporting guideline.

## 3. Results

### 3.1. Demographics and Baseline Comorbidities

From 2016 through 2022, 11,158,836 index HF hospitalizations met inclusion criteria, of which 32,419 (0.29%) had a secondary diagnosis of RSV (HF-RSV). These were propensity matched 1:1 to 32,419 HF hospitalizations without RSV (HF-noRSV). Baseline characteristics before and after matching are summarized in Table 1.

Before matching, HF-RSV hospitalizations had more representation from the highest household income quartile (25.1% vs. 19.1%) and less from the lowest quartile (22.1% vs. 29.6%) compared with HF-noRSV (SMD = 0.20). Chronic lung disease was more prevalent in HF-RSV (55.9% vs. 36.6%; SMD = 0.39). The distribution of HF-RSV hospitalizations shifted over time, increasing from 6.9% in 2016 to 19.3% in 2020, followed by decline in 2021 (8.1%) and increase again in 2022 (15.4%).

The median age of HF-RSV patients was 77 years (IQR: 67–86), and 57.9% were women. Comorbid conditions were common, including hypertension (89.3%), chronic lung disease (55.9%), dyslipidemia (52.6%), diabetes (43.9%), chronic kidney disease (42.7%), and coronary artery disease (39.6%). Most HF-RSV hospitalizations occurred at large (58.5%), urban (87.7%), and teaching hospitals (74.2%). After matching, all covariates were well balanced (SMD < 0.10).

### 3.2. Outcomes

In the propensity-matched cohort, HF-RSV hospitalizations had higher in-hospital mortality (aOR: 1.12; 95% CI: 1.04–1.20; *p* = 0.001) and greater odds of septic shock, acute respiratory failure, and need for noninvasive mechanical ventilation (all *p* < 0.001).

In contrast, HF-RSV was associated with lower odds of cardiogenic shock, ventricular tachycardia or fibrillation, ischemic stroke, and transient ischemic attack (all *p* ≤ 0.001). Thirty-day all-cause readmissions were also significantly lower among HF-RSV hospitalizations (aOR: 0.54; 95% CI: 0.46–0.56; *p* < 0.001).

There were no significant differences between groups in the odds of acute coronary syndrome, cardiac arrest, invasive mechanical ventilation, acute renal failure, or discharge disposition (Table 2).

### 3.3. Healthcare Resource Utilization

HF-RSV hospitalizations were associated with greater healthcare resource use. Median hospitalization cost was modestly higher in the HF-RSV group compared with HF-noRSV ($14,637.82 [IQR: $8425.09–$24,682.50] vs. $14,384.85 [IQR: $9042.18–$23,633.10]). In adjusted models, RSV infection was associated with an estimated 2% higher hospitalization cost (adjusted coefficient: 0.02; 95% CI: 0.01–0.02; *p* < 0.001). Median length of stay was longer among HF-RSV hospitalizations (6 days [IQR: 3–9] vs. 4 days [IQR: 3–8]), and RSV infection was associated with a 14% longer LOS in adjusted Poisson regression (adjusted coefficient: 0.14; 95% CI: 0.13–0.14; *p* < 0.001). The 30-day readmissions were lower in HF-RSV (aOR: 0.54; 95% CI: 0.46–0.56; *p* < 0.001) (Table 2).

## 4. Discussion

In this large, contemporary analysis of HF hospitalizations across multiple US states, 32,419 (0.29%) had a concomitant diagnosis of RSV. HF hospitalizations with RSV (HF-RSV) were associated with higher in-hospital mortality, greater odds of septic shock, more than threefold higher odds of acute respiratory failure, and approximately twofold higher odds of noninvasive mechanical ventilation compared with matched HF hospitalizations without RSV (HF-noRSV), as well as longer length of stay (LOS) and slightly higher hospitalization costs. Conversely, HF-RSV was associated with lower odds of cardiogenic shock, ventricular arrhythmias, ischemic stroke, TIA, and lower 30-day readmissions.

The complication profile observed in HF-RSV is consistent with the biological effects of RSV as a primarily respiratory infectious pathogen, with predominance of acute respiratory failure and septic shock, whereas traditional cardiovascular and cerebrovascular events characterized HF-noRSV admissions. We hypothesize that management considerations (not available in NRD) may also influence our findings. Cardiovascular and cerebrovascular complications have established evidence-based treatments, whereas therapy for RSV in adults is largely supportive, and current guidelines do not recommend routine antiviral therapy [16]. The absence of targeted therapeutics may partly explain the higher mortality and greater resource use observed in HF-RSV. In contrast, those who survive the acute event to hospital discharge may return to baseline HF trajectory after resolution of acute RSV infection, leading to lower 30-day readmission rates. Survival bias is also likely, because higher in-hospital mortality among HF-RSV hospitalizations reduces the number of patients eligible for early readmission.

The temporal distribution of HF-RSV hospitalizations in our study—an increase from 2016 through 2020, a nadir in 2021, and a rebound in 2022—likely reflects both evolving testing practices and pandemic-related changes in viral circulation. The overall rise over time is consistent with increased awareness and broader availability of RSV testing in adults. The decline in 2021 aligns with reports of reduced RSV activity during the COVID-19 pandemic, attributed to nonpharmacologic public health measures; and the subsequent resurgence in 2022 parallels the relaxation of mitigation measures and the well-described rebound in RSV circulation [17]. Chronic lung disease was more common among HF-RSV hospitalizations before matching in our analysis, which is in keeping with non-HF cohorts in which patients with COPD and other chronic pulmonary conditions are overrepresented among RSV cases [18,19].

The prevalence of RSV in HF hospitalizations in our US cohort (0.29%) was lower than the 0.47% reported in a recent national Spanish analysis of HF hospitalizations from 2018–2022 [10]. Differences in testing thresholds, RSV circulation patterns, and healthcare systems between Spain and the US may contribute to this discrepancy. In addition, we excluded hospitalizations with COVID-19 or influenza co-infections, which occur in overlapping seasons and have been associated with adverse outcomes among HF hospitalizations [20,21]. This exclusion likely lowered the proportion of HF admissions categorized as HF-RSV in our study.

Our findings are concordant with prior work showing that RSV is associated with substantial morbidity and mortality in non-HF populations. In a large Kaiser Permanente cohort, RSV compared with influenza was associated with longer LOS, greater critical care use, and higher 1-year mortality [18]. Another multicenter study of adults hospitalized with acute respiratory illness showed that RSV, relative to influenza, was associated with longer hospitalization and increased need for mechanical ventilation [19]. However, data specifically focusing on HF hospitalizations have been limited. A prior National Inpatient Sample analysis of HF hospitalizations between 2016 and 2019 reported that RSV was not associated with in-hospital mortality [8]. Differences between that study and ours—including the inclusion of RSV pneumonia ICD-10 codes in our RSV definition and the extension of follow-up through 2022, encompassing evolving testing practices and pandemic-related changes—may partly account for the higher mortality observed in our analysis. The recent Spanish HF-RSV study reported higher in-hospital mortality (10.16%), greater use of noninvasive ventilation (9.46%), and longer LOS, as well as lower rates of myocardial infarction and cerebrovascular disease in RSV-positive HF hospitalizations [10]. These associations are broadly consistent with our findings, despite differences in absolute event rates and costs, which may reflect differences in patient populations, coding practices, inclusion of COVID-19 and influenza co-infections, healthcare delivery, and reimbursement structures. Our study findings also align with previous studies on RSV outcomes when HF is complicated by RSV, based on US cohorts. A prior investigation involving 2042 hospitalized RSV cases through the RSV Hospitalization Surveillance Network (RSV-NET) from 2015 to 2017 estimated that 577 patients had heart failure (HF). Those RSV cases with HF experienced increased mortality, more frequent ICU admissions, and longer hospital stays [9]. A separate RSV-NET study of 6248 RSV hospitalizations among adults aged 50 and above found that acute heart failure (965 cases) was linked to a higher risk of in-hospital death and the need for invasive mechanical ventilation in RSV patients [4].

The lower rates of adverse cardiovascular events observed in HF-RSV must be interpreted with caution. Rather than indicating a protective effect of RSV, these findings are more likely attributable to competing risks, in which critical respiratory illness and mortality supersede or precede the occurrence, detection, reporting and documentation of cardiovascular events. Therefore, lower observed rates of cardiovascular events in the HF-RSV group should not be interpreted as evidence of lower cardiovascular risk.

To our knowledge, this is the most extensive study to date evaluating RSV in HF hospitalizations, leveraging a large, multi-state, nationwide dataset of more than 11 million HF hospitalizations from 2016–2022, including 32,419 with RSV. The breadth of the NRD allowed us to examine a wide range of clinical outcomes and healthcare utilization metrics in a rigorously matched cohort. Collectively, our findings highlight the importance of recognizing RSV as a clinically relevant comorbidity in HF hospitalizations. They also underscore the need for strategies to improve RSV prevention, diagnosis, and management in patients with HF, including evaluation of the impact of recently approved RSV vaccines in this high-risk population and the development of effective antiviral therapies.

This study has several limitations. First, its retrospective observational design precludes causal inference regarding the relationship between RSV infection and outcomes in HF hospitalizations. Second, the NRD lacks detailed clinical information, including HF phenotype or ejection fraction, New York Heart Association functional class, guideline-directed medical therapy, hemodynamics, and RSV-specific laboratory data. As a result, residual confounding and unmeasured differences in disease severity or management may persist despite propensity matching. Third, our definition of RSV relied on ICD-10 codes. This introduces exposure misclassification, as RSV testing is not universal in adult HF hospitalizations. Patients with mild or untested RSV may have been misclassified into the control group, potentially biasing results toward the null. Fourth, by restricting our analysis to hospitalizations with a primary diagnosis of HF, we may have introduced collider bias by conditioning on decompensated heart failure as a collider, thereby distorting the observed associations between RSV and outcomes. Accordingly, these findings should be interpreted as associations observed in HF hospitalizations rather than as risk estimates for RSV patients. Fifth, outcomes were limited to in-hospital events and 30-day readmissions within the same calendar year; longer-term consequences of RSV in patients with HF could not be assessed. Sixth, because the NRD is restricted to US hospitalizations, generalizability to other countries and outpatient settings may be limited. Seventh, comorbidities and outcomes were identified using ICD-10-CM/PCS codes, which are subject to coding variability and may underestimate chronic conditions that were not documented during the index admission. Eighth, interpretation of 30-day readmission findings requires caution. Higher in-hospital mortality among HF-RSV hospitalizations reduces the number of individuals eligible for readmission, introducing survival bias. Furthermore, because NRD does not allow year-to-year linkage of patients, data were interpreted at the hospitalization level rather than the patient level. Pursuant to the same structural limitation, we excluded December discharges to preserve the 30-day follow-up window, removing a substantial portion of hospitalizations that occur during the peak RSV season. This likely leads to underestimation of true readmission rates in the RSV group.

## 5. Conclusions

In a large, contemporary cohort of US HF hospitalizations, RSV infection was associated with higher in-hospital mortality, greater odds of septic shock and acute respiratory failure, increased use of noninvasive mechanical ventilation, and greater healthcare resource utilization, including longer hospital stay and higher hospitalization costs. These findings emphasize the importance of assessing the impact of preventive and therapeutic strategies in this vulnerable population in future studies.

## 6. Clinical Perspective

In heart failure hospitalizations, concomitant RSV infection is associated with higher in-hospital mortality, increased risk of septic shock and acute respiratory failure, greater use of noninvasive mechanical ventilation, and higher healthcare resource utilization, including longer hospitalization and increased costs. These findings underscore association of RSV with acute clinical decompensation in HF. Further investigation is needed to determine whether recently approved RSV vaccines can reduce severe outcomes in this high-risk population and to clarify the potential role of targeted antiviral therapies and standardized testing strategies in improving prognosis.

## Figures and Tables

**Table 1:** Baseline characteristics of Heart Failure hospitalizations stratified by Respiratory Syncytial Virus diagnosis.

Characteristic ^a^—*n* (%)	Unweighted Heart Failure Hospitalizations			1:1 Propensity Score Matching		
	With Respiratory Syncytial Virus (n = 32,419)	Without Respiratory Syncytial Virus (n = 11,126,417)	SMD ^b^	With Respiratory Syncytial Virus (n = 32,419)	Without Respiratory Syncytial Virus (n = 32,419)	SMD ^b^
**Patient characteristics**						
Age (years)—Median (IQR)	77 (67–86)	74 (64–83)	0.17	77 (66–86)	77 (66–85)	0.02
Women	18,791 (57.9)	5,414,855 (48.7)	0.18	18,722 (57.7)	18,869 (58.2)	0.009
Primary expected payer						
Medicare	26,223 (80.9)	8,428,605 (75.8)	0.12	26,219 (80.9)	26,607 (82.1)	0.02
Medicaid	2362 (7.3)	960,607 (8.6)		2362 (7.3)	2289 (7.1)	
Private	2938 (9.1)	1,256,966 (11.3)		2938 (9.1)	2678 (8.3)	
Uninsured	345 (1.1)	194,033 (1.7)		345 (1.1)	330 (1.1)	
Others	477 (1.5)	250,834 (2.3)		475 (1.5)	485 (1.5)	
Median household income, percentile						
0–25th	7087 (22.1)	3,254,594 (29.6)	0.20	7087 (22.1)	7640 (23.6)	0.004
26th–50th	8379 (26.1)	2,995,593 (27.3)		8379 (26.1)	8145 (25.1)	
51st–75th	8610 (26.8)	2,638,437 (24.1)		8610 (26.8)	8577 (26.5)	
76th–100th	8035 (25.1)	2,099,289 (19.1)		8035 (25.1)	8057 (24.8)	
Years						
2016	2252 (6.9)	1,415,985 (12.7)	0.06	2265 (6.9)	3472 (10.7)	0.01
2017	4039 (12.5)	1,583,518 (14.2)		4039 (12.5)	4295 (13.3)	
2018	6105 (18.8)	1,625,143 (14.6)		6105 (18.8)	4888 (15.1)	
2019	6149 (18.9)	1,738,955 (15.6)		6129 (18.9)	5354 (16.5)	
2020	6248 (19.3)	1,572,885 (14.1)		6248 (19.3)	4990 (15.4)	
2021	2628 (8.1)	1,587,494 (14.3)		2628 (8.1)	4421 (13.6)	
2022	4998 (15.4)	1,602,439 (14.4)		4998 (15.4)	4999 (15.4)	
Comorbidities						
Smoking	9407 (29.1)	2,990,195 (26.9)	0.04	9407 (29.1)	9166 (28.3)	0.01
Dyslipidemia	17,055 (52.6)	6,088,257 (54.7)	0.04	17,055 (52.6)	17,061 (52.6)	0.001
Diabetes	14,221 (43.9)	4,951,416 (44.5)	0.01	14,221 (43.9)	14,184 (43.7)	0.005
Hypertension	28,942 (89.3)	9,973,032 (89.6)	0.01	28,942 (89.3)	29,021 (89.5)	0.008
Obesity	8016 (24.7)	2,702,038 (24.3)	0.01	8016 (24.7)	7892 (24.3)	0.009
Known CAD	12,841 (39.6)	4,854,377 (43.6)	0.08	12,841 (39.6)	12,524 (38.6)	0.01
Prior MI	3694 (11.4)	1,433,648 (12.8)	0.04	3694 (11.4)	3564 (10.9)	0.01
Prior PCI	3131 (9.7)	1,289,404 (11.6)	0.06	3131 (9.7)	2986 (9.2)	0.01
Prior CABG	3064 (9.5)	1,173,948 (10.6)	0.03	3064 (9.5)	2954 (9.1)	0.01
Prior TIA/stroke	3025 (9.3)	1,137,040 (10.2)	0.03	3025 (9.3)	2897 (8.9)	0.01
Peripheral vascular disease	4893 (15.1)	1,898,318 (17.1)	0.05	4893 (15.1)	4733 (14.6)	0.01
Anemia	2137 (6.6)	787,119 (7.1)	0.01	2137 (6.6)	1986 (6.1)	0.01
Chronic kidney disease	13,840 (42.7)	4,517,728 (40.6)	0.04	13,840 (42.7)	13,574 (41.9)	0.01
Chronic lung disease	18,118 (55.9)	4,073,933 (36.6)	0.39	18,118 (55.9)	17,768 (54.8)	0.01
Chronic liver disease	1638 (5.1)	739,473 (6.6)	0.06	1638 (5.1)	1547 (4.8)	0.01
Coagulopathy	3365 (10.4)	1,145,555 (10.3)	0.05	3365 (10.4)	3233 (9.9)	0.01
Hypothyroidism	6890 (21.3)	2,048,329 (18.4)	0.07	6890 (21.3)	6698 (20.7)	0.01
Pulmonary circulation disorders	6495 (20.1)	1,851,481 (16.6)	0.08	6495 (20.1)	6068 (18.7)	0.03
Cancer	465 (1.4)	270,677 (2.4)	0.07	465 (1.4)	426 (1.3)	0.01
Elixhauser comorbidity Index, median (IQR)	17 (13–23)	17 (12–22)	0.13	17 (13–23)	17 (13–23)	0.04
**Hospital characteristics**						
Bed size						
Small	5768 (17.8)	1,954,441 (17.6)	0.05	5768 (17.8)	5160 (15.9)	0.01
Medium	7679 (23.7)	3,137,664 (28.2)		7679 (23.7)	8704 (26.8)	
Large	18,972 (58.5)	6,034,314 (54.2)		18,972 (58.5)	18,555 (57.2)	
Location						
Urban	28,346 (87.7)	9,282,547 (83.7)	0.11	28,346 (87.7)	28,528 (88)	0.008
Rural	3964 (12.3)	1,803,110 (16.3)		3964 (12.3)	3891 (12)	
Teaching Status						
Nonteaching	6192 (19.1)	2,415,674 (21.7)	0.01	6192 (19.1)	6236 (19.3)	0.01
Teaching	24,050 (74.2)	7,760,495 (69.8)		24,050 (74.2)	23,803 (73.4)	

^a^ Represented as number (percentage) or median (interquartile range). ^b^ A standardized mean difference of 0.2 to 0.5, 0.5 to 0.8, and >0.8 represents small, medium, and large effect sizes, respectively. Abbreviations: CABG: coronary artery bypass graft; CAD: coronary artery disease; IQR: interquartile range; MI: myocardial infarction; PCI: percutaneous coronary intervention, SMD: standardized mean difference; TIA: transient ischemic attack.

**Table 2:** Association between Respiratory Syncytial Virus and in-hospital outcomes in Heart Failure hospitalizations.

**Outcomes ^a^**	**With Respiratory Syncytial Virus** **(n = 32,419)**	**Without Respiratory Syncytial Virus** **(n = 32,419)**	**Odds Ratio (95% Confidence Interval)**		***p*-Value**
			**Unadjusted**	**Adjusted ^b^**	
In-hospital outcomes					
Mortality	1809 (5.6)	1638 (5.1)	1.11 (1.03–1.18)	1.12 (1.04–1.20)	0.001
Septic shock	1639 (5.1)	1194 (3.7)	1.39 (1.29–1.50)	1.40 (1.29–1.52)	<0.001
Cardiogenic shock	601 (1.9)	690 (2.1)	0.86 (0.77–0.97)	0.82 (0.73–0.92)	0.001
Acute coronary syndrome	3334 (10.3)	3340 (10.3)	0.99 (0.94–1.04)	0.99 (0.94–1.04)	0.70
Ventricular tachycardia/Ventricular fibrillation	1371 (4.2)	1855 (5.7)	0.72 (0.67–0.78)	0.73 (0.68–0.78)	<0.001
Cardiac arrest	451 (1.4)	506 (1.6)	0.88 (0.78–1.01)	0.90 (0.79–1.03)	0.13
Acute respiratory failure	19,374 (59.8)	10,273 (31.7)	3.20 (3.10–3.30)	3.43 (3.32–3.55)	<0.001
Invasive mechanical ventilation	160 (0.5)	156 (0.5)	1.02 (0.82–1.27)	1.04 (0.83–1.30)	0.72
Noninvasive mechanical ventilation	5119 (15.8)	2618 (8.1)	2.13 (2.03–2.24)	2.15 (2.04–2.26)	<0.001
Ischemic stroke	273 (0.8)	850 (2.6)	0.31 (0.27–0.36)	0.31 (0.27–0.36)	<0.001
Transient ischemic attack	73 (0.2)	213 (0.7)	0.34 (0.26–0.44)	0.33 (0.25–0.44)	<0.001
Acute renal failure	10,489 (32.4)	10,574 (32.6)	0.98 (0.95–1.02)	0.96 (0.93–1.01)	0.06
Discharge to SNF	7913 (24.4)	7989 (24.6)	0.98 (0.95–1.02)	0.97 (0.94–1.01)	0.27
30-day readmission	2827 (8.7)	4654 (14.4)	0.56 (0.54–0.59)	0.54 (0.46–0.56)	<0.001
**Outcomes**	**With Respiratory Syncytial Virus** **(n = 32,419)**	**Without Respiratory Syncytial Virus** **(n = 32,419)**	**Coefficient (95% confidence interval)**		***p*** **-Value**
			**Unadjusted**	**Adjusted**	
Cost	14,637.82 (8425.09–24,682.5)	14,384.85 (9042.18–23,633.1)	0.2 (0.2–0.3)	0.02 (0.01–0.02)	<0.001
Length of stay	6 (3–9)	4 (3–8)	0.15 (0.14–0.15)	0.14 (0.13–0.14)	<0.001

^a^ Represented as Frequency (percentage) or median (interquartile range). After 1:1 propensity-matching, the association between Respiratory Syncytial Virus and in-hospital outcomes was assessed using multivariate regression. *p*-value < 0.05 was considered significant. ^b^ Baseline characteristics in Table 1 were used for adjusted analysis. SNF: skilled nursing facility.

## Data Availability

The data used in this study are from the Nationwide Readmissions Database (NRD), Healthcare Cost and Utilization Project (HCUP), Agency for Healthcare Research and Quality. These data are available from HCUP but restrictions apply to their availability, which were used under license for the current study and are not publicly available. Data are, however, available from the authors upon reasonable request and with permission of HCUP.

## Supplementary Materials

The following supporting information can be downloaded at: https://www.mdpi.com/article/10.3390/jcm15030990/s1, Supplemental Table S1: International Classification of Diseases, Tenth Revision, Clinical Modification and Procedure Coding System (ICD-10-CM/PCS) codes used to identify baseline comorbidities and procedures.

## Abbreviations

aOR	adjusted odds ratio
CI	confidence interval
HF	heart failure
HF-RSV	heart failure hospitalizations with RSV
HF-noRSV	heart failure hospitalizations without RSV
IQR	interquartile range
LOS	length of stay
NRD	Nationwide Readmissions Database
RSV	Respiratory Syncytial Virus
TIA	Transient Ischemic Attack
US	United States
