# Supplementary material for: Comparison of Heart Failure Hospitalizations with and Without Respiratory Syncytial Virus: A Nationwide Administrative Data Analysis
Source: J Clin Med. 2026 Jan 26;15(3):990. doi: 10.3390/jcm15030990 (PMC12898105; doi:10.3390/jcm15030990)
Supplement: Supplementary file 1 [file jcm-15-00990-s001.zip › jcm-4099461-supplementary.pdf]

Supplemental Methods:

Supplemental Table S1: International Classification of Diseases, Tenth Revision, Clinical Modification and Procedure Coding System (ICD-10-CM/PCS) codes used to identify baseline comorbidities and procedures.

Variable	*ICD-10-CM/PCS or Elixhauser comorbidities software**
RSV	B974, J121, J205, J210
Influenza	J09, J09.X,J09.X1, J09.X2, J09.X3, J09.X9, J10, J10.0, J10.00, J10.01, J10.08, J10.1, J10.2, J10.8, J10.81, J10.82, J10.83, J10.89, J11,J11.0, J11.00, J11.08, J11.1, J11.2, J11.8, J11.81, J11.82, J11.83, J11.89
Covid-19	U07.1
Heart failure	I50 (all I50)
Diabetes mellitus	Elixhauser comorbidities software
Smoking	Z72.0, Z87.891, F17 (all), O99.33x
Dyslipidemia	Elixhauser comorbidities software
Hypertension	Elixhauser comorbidities software
Obesity	Elixhauser comorbidities software
Coagulopathy	Elixhauser comorbidities software
Known CAD	I25.xxx (except I25.2)
Prior MI	I25.2
Prior PCI	Z95.5, Z98.61
Prior CABG	Z95.1
Prior TIA/Stroke	Elixhauser comorbidities software
Peripheral vascular disease	Elixhauser comorbidities software
Chronic kidney disease	Elixhauser comorbidities software
Chronic pulmonary disease	Elixhauser comorbidities software
Chronic liver disease	Elixhauser comorbidities software

Anemia	Elixhauser comorbidities software
Hypothyroidism	Elixhauser comorbidities software
Pulmonary Circulation Disorders	Elixhauser comorbidities software
Cancer	Elixhauser comorbidities software
**Outcomes**	
Acute Renal Failure	All N17
Invasive Mechanical Ventilation	0BH17EZ, 0BH18EZ, 5A1935Z, 5A1945Z, 5A1955Z
Noninvasive Mechanical Ventilation	5A09357, 5A09457, 5A09557, 5A09358, 5A09458, 5A09558, 5A0935Z, 5A0945Z, and 5A0955Z
Cardiogenic Shock	R570
Ventricular Tachycardia	All I472
Septic Shock	R6521
Acute respiratory failure	All J96
Acute coronary syndrome	I21x (All I21)
Ischemic Stroke	G46.3, G46.4, G46.5, G46.6, G46.7, I63.00, I63.011, I63.012, I63.013, I63.019, I63.02, I63.031, I63.032, I63.033, I63.039, I63.09, I63.10, I63.111, I63.112, I63.113, I63.119, I63.12, I63.131, I63.132, I63.133, I63.139, I63.19, I63.20, I63.211, I63.212, I63.213, I63.219, I63.22, I63.231, I63.232, I63.233, I63.239, I63.29, I63.30, I63.311, I63.312, I63.313, I63.319, I63.321, I63.322, I63.323, I63.329, I63.331, I63.332, I63.333, I63.339, I63.341, I63.342, I63.343, I63.349, I63.39, I63.40, I63.411, I63.412, I63.413, I63.419, I63.421, I63.422, I63.423, I63.429, I63.431, I63.432, I63.433, I63.439, I63.441, I63.442, I63.443, I63.449, I63.49, I63.50, I63.511, I63.512, I63.513, I63.519, I63.521, I63.522, I63.523, I63.529, I63.531, I63.532, I63.533, I63.539, I63.541, I63.542, I63.543, I63.549, I63.59, I63.6, I63.81, I63.89, I63.9
Ventricular fibrillation	I49
Cardiac Arrest	All I46

TIA	All G45

Abbreviations: CABG, coronary artery bypass graft; CAD, coronary artery disease; MI, myocardial infarction; PCI, percutaneous coronary intervention; RSV, respiratory syncytial virus; TIA, transient ischemic attack.

*Comorbidities identified from the Elixhauser Comorbidity Software included in the Nationwide Readmissions Database
